# Neutrophil-to-albumin ratio: a novel predictor of osteoporosis in rheumatoid arthritis

**DOI:** 10.3389/fimmu.2025.1666884

**Published:** 2025-09-17

**Authors:** Yifang Zhang, Zhongyu He, Kaiqiang Li, Qiuping Wu, Shigang Wang, Minying Liu, Qingping Liu, Qiang Xu, Xiangying Kong, Changsong Lin

**Affiliations:** ^1^ The First Clinical Medical School, Guangzhou University of Chinese Medicine, Guangzhou, China; ^2^ China Joint Graduate School of Traditional Chinese Medicine, Suzhou, China; ^3^ Department of Rheumatology, The First Affiliated Hospital of Guangzhou University of Chinese Medicine, Guangzhou, China; ^4^ Institute of Chinese Materia Medica, China Academy of Chinese Medical Sciences, Beijing, China

**Keywords:** rheumatoid arthritis, osteoporosis, neutrophil percentage to albumin ratio, inflammation, National Health and Nutrition Examination Survey, risk stratification

## Abstract

**Background:**

Osteoporosis (OP) frequently coexists with rheumatoid arthritis (RA), but validated predictors of early risk are not extensively studied. This study seeks to examine the relationship between the neutrophil percentage-to-albumin ratio (NPAR) and the likelihood of developing RA-related OP(RA-OP).

**Methods:**

After investigating the relationship between the NPAR and RA-OP in the clinical retrospective study, we further validated this association using data from the National Health and Nutrition Examination Survey (NHANES) database (2005-2020 cycles). This retrospective study enrolled 718 RA patients from the Rheumatology Department of the First Affiliated Hospital of Guangzhou University of Chinese Medicine between January 2020 and December 2024. Patients were categorized into low-NPAR (<1.7598) and high-NPAR (≥1.7598) groups based on the median NPAR. Extracted clinical data encompassed demographic characteristics, comorbidities, serological markers, and other laboratory parameters. Preliminary univariate and multivariate logistic regression analyses assessed potential associations between NPAR and RA-OP, multi-model adjusted logistic regression was subsequently applied to evaluate the independent association, subgroup analyses examined consistency across demographic and clinical strata, Receiver operating characteristic (ROC) curve analysis assessed NPAR’s diagnostic performance, and then Restricted cubic splines (RCS) visualized potential non-linear relationships. Finally using the identical statistical framework, we validated findings within the NHANES cohort.

**Results:**

The high-NPAR group exhibited significantly higher OP incidence than the low-NPAR group (39.0% vs. 26.5%; P<0.001). After full adjustment (Model 4), NPAR remained independently associated with increased RA-OP risk as a categorical variable (high vs. low NPAR: adjusted OR = 1.70 (95%CI: 1.01~2.88); P = 0.049). Subgroup analyses demonstrated no significant interaction effects (P-interaction>0.05) except for disease duration. The ROC curve showed an Area Under the Curve(AUC) of 0.58 (95%CI: 0.53~0.63) and NPAR cut-off of 1.886. The covariate-adjusted RCS indicated a linear dose-response relationship (P overall=0.033; P nonlinearity=0.168). NHANES cohort analysis independently validated both the NPAR-RA-OP association and its linear characteristic.

**Conclusion:**

NPAR, serving as a novel composite biomarker integrating neutrophil-mediated inflammation and nutritional status (via albumin), independently predicts OP risk in RA. Its derivation from routine clinical parameters renders NPAR a readily deployable, cost-effective tool for OP risk stratification in clinical practice.

## 1 Introduction

OP is a chronic systemic bone disease leading to increased bone fragility and fracture risk, characterized by a T-score≤-2.5 standard deviations compared to young adults. Patients with inflammatory rheumatism and musculoskeletal disorders (iRMDs) exhibit a higher risk of OP compared to non-iRMD individuals. Among them, patients with RA have a fracture risk twice that of normal individuals (1), with fragility fractures a significant comorbidity in RA (2). About 30% to 50% of RA patients have concurrent OP, and the risk of RA-OP is closely associated with disease duration, disease activity, age, gender, local and systemic inflammation, and glucocorticoid (GC) use, among which systemic inflammation and GC therapy play a major role (1–3).Poor functional status and frailty constitute another important risk factor for low bone mineral density and fractures in RA patients (4).

RA patients commonly exhibit malnutrition-related conditions such as anemia (5), hypoalbuminemia (6), low body mass index (BMI) (7), and sarcopenia (8), and all of which are linked to OP (9–12). Additionally, reduced bone mass and OP are identified as independent predictors of atlantoaxial subluxation in individuals with low BMI (13). While prior studies have mainly concentrated on inflammatory markers, disease activity, medication usage, and patient demographics like gender, disease duration, smoking, and alcohol consumption in assessing OP risk in RA patients, few have thoroughly examined this risk by considering both inflammatory and nutritional statuses. NPAR is a clinically accessible metric that integrates inflammation and nutritional status, calculated as the ratio of neutrophil percentage to serum albumin. Previous research indicates that elevated NPAR levels are associated with an increased risk of RA (14), yet its association with OP risk in RA patients remains unexplored. This study seeks to explore the correlation between NPAR and RA-OP.

The N-terminal propeptide of type I procollagen (PINP) and the C-terminal telopeptide of type I collagen (CTX-I) are established bone turnover markers in clinical practice, reflecting bone formation and resorption, respectively (15). However, these markers are susceptible to various confounding factors such as dietary intake and recent fracture history. Importantly, they are not suitable for OP diagnosis, and neither improve the predictive capacity for individual bone loss or fracture risk (16). Early risk factors for OP in RA patients remain inadequately explored. Prompt recognition of these factors can facilitate tailored management strategies and potentially reduce the prevalence of OP and fragility fractures in this specific population.

Since patients with RA frequently exhibit both elevated inflammatory levels and poor nutritional status, which significantly contribute to the development of OP. This study investigated the association between NPAR, a biomarker that integrates both nutritional and inflammatory status, and RA-related OP, rather than more established indicators such as neutrophil-to-lymphocyte ratio (NLR) for immune evaluation or systemic immune-inflammation index (SII) for systemic inflammation assessment. Our study examined clinical data from RA patients to investigate the OP incidence in relation to levels of NPAR. The relationship between NPAR levels and OP was evaluated through multivariable logistic regression analysis with progressively adjusted models, subgroup analyses, and non-linearity testing. External validation of the results was conducted using the NHANES 2005-2020 cohort. The findings indicate that higher NPAR levels are independently associated with increased OP risk in individuals with RA.

## 2 Method

### 2.1 Study population

**Figure 1:**
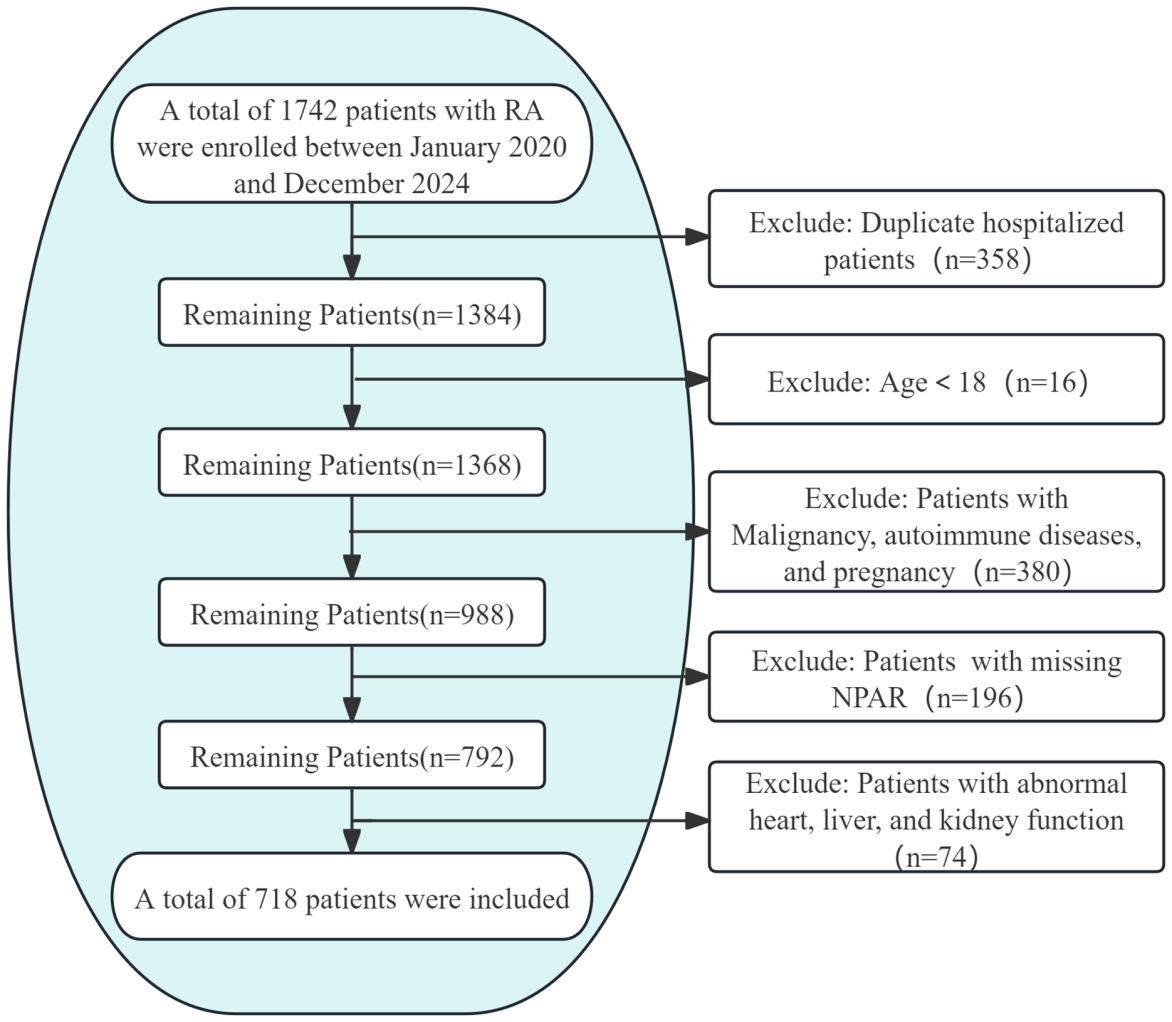
Flow Diagram of Study Population Selection.

This cross-sectional study analyzed clinical data from RA patients admitted to the Rheumatology Department of the First Affiliated Hospital of Guangzhou University of Chinese Medicine between January 2020 and December 2024. Approval for the study protocol was obtained from the Medical Ethics Committee of Guangzhou University of Chinese Medicine (Approval No.: JY2025-088). Out of 1,742 hospitalizations with a primary RA diagnosis during the study period, only data from the initial admission within this timeframe were considered for patients with multiple admissions. Inclusion criteria comprised meeting the 2010 ACR/EULAR classification criteria for RA and being aged 18 years or older. Exclusion criteria included incomplete OP diagnostic data, missing NPAR values, comorbid malignancies, autoimmune diseases other than RA, pregnancy, heart failure, liver/renal function indicators exceedingly twice the upper limit of normal, and a maximum of 10% missing data points. Following the application of these criteria, 718 participants aged between 18 and 85 were included in the study (**Figure 1**). For variables with missing data below 10%, categorical variables were denoted as “unrecorded,” while multiple imputation was performed for continuous variables.

### 2.2 Diagnosis of OP

The diagnosis of OP was established through the review of clinical records and bone mineral density (BMD) assessments conducted during the patient’s hospital stay. BMD findings were classified based on T-scores as follows: Normal bone density: T-score≥-1; Osteopenia: -2.5≤T-score<-1; Osteoporosis: T-score<-2.5.

### 2.3 Definition and stratification of NPAR

The NPAR was computed by dividing the neutrophil percentage by the serum albumin level, resulting in a continuous variable. Patients were categorized into high- and low-NPAR groups based on the cohort median (1.7598).

### 2.4 Demographic and clinical characteristics

Demographic and clinical data included age, gender, disease duration (<1 year, 1˜5 years, 6˜10 years, 11˜20 years, >20 years), body mass index [underweight (<18.5 kg/m²), normal weight (18.5˜24.99 kg/m²), overweight (25.0˜29.99 kg/m²), obesity (≥30.0 kg/m²)], smoking status (current smoker/non-smoker), and alcohol consumption (current consumer/non-consumer).

### 2.5 Comorbidities

Comorbidities assessed in the study encompassed a range of conditions such as diabetes mellitus, hypertension, cardiovascular diseases (CVDs), interstitial lung disease (ILD), dyslipidemia, anemia, OP, hyperuricemia/gout, thyroid disorders, and infectious diseases (including pulmonary and/or urinary tract infections, influenza A, Helicobacter pylori infection, hepatitis B, and pulmonary tuberculosis). CVDs were examined as composite events, including coronary artery disease, valvular heart disease, angina pectoris, and cardiac arrhythmias.

### 2.6 Laboratory examination

The laboratory test results comprised the following parameters: C-reactive protein (CRP, reference range:0˜8 mg/L), Erythrocyte sedimentation ate (ESR, female: 0˜20mm/h, male:0˜15mm/h), Anti-citrullinated protein antibody (ACPA, normal: 0˜5U/mL; values exceeding 200 U/mL were classified as the “>200 group”), Rheumatoid factor (RF, 0˜20 IU/mL), White blood cell count (WBC, 4˜10×10^9^/L), Lymphocyte count (LYM, 1.6˜4×10^9^/L),Neutrophil count (NEU, 2˜7.5×10^9^/L), NEU percentage, Red blood cell count (RBC, female: 3.5˜5×10^12^/L, male: 4.5˜5×10^12^/L),Hemoglobin (HGB, female: 110˜150g/L, male: 120˜160 g/L), Platelet count (PLT, 100˜300×10^9^/L), D-dimer (DDi, 0˜0.55mg/L), Albumin (ALB, 40˜55 g/L), Total cholesterol (TC, 2.6˜5.2mmol/L), Triglycerides (TG, 0.34˜1.7mmol/L), Low-density lipoprotein (LDL, ≤3.37mmol/L),High-density lipoprotein (HDL, >1.04mmol/L), and NPAR.

### 2.7 Medication use

We collected medication information during hospitalization, including: nonsteroidal anti-inflammatory drugs (NSAIDs), methotrexate (MTX), leflunomide (LEF), hydroxychloroquine (HCQ), sulfasalazine (SSZ), iguratimod (IGU), glucocorticoids (GCs), tofacitinib, baricitinib, and tumor necrosis factor-alpha inhibitors (TNF-α inhibitors).

### 2.8 Statistical analysis

Descriptive analyses presented normally distributed continuous variables as mean (standard deviation, SD) and group comparison using independent t-tests. Non-normally distributed continuous variables were expressed as median (interquartile range, IQR) and analyzed with Mann-Whitney U tests. Categorical variables were shown as n (%) and assessed using chi-square tests. The association between NPAR and RA-OP was examined through multivariable logistic regression and nonlinear trend analysis. Four logistic regression models were constructed with increasing covariate adjustments: Model 1 was unadjusted, Model 2 included demographic and lifestyle factors (age, gender, disease duration, BMI, smoking status, and alcohol consumption), Model 3 added comorbidities (ILD, CVDs, dyslipidemia, anemia, diabetes, hypertension, thyroid disorders, hyperuricemia/gout, and infectious diseases), and Model 4 further included laboratory parameters (RF, ACPA, CRP, ESR, WBC, RBC, HGB, NEU, PLT, LYM, DDi, TC, TG, HDL, and LDL) and GCs. Stratified analyses were conducted to assess the impact of clinical confounders on OP development. RCS were utilized to explore potential nonlinear relationships between NPAR and OP in RA. All analyses were carried out using R version 4.3.3 (R Foundation for Statistical Computing, Vienna, Austria), with statistical significance set at a two-sided P value<.05.

## 3 Results

### 3.1 Patient baseline characteristics

Given the inconsistent NPAR cutoff values in literature (17, 18), we used the median NPAR value of 1.7598 (19)to divide the 718 RA patients included in the study into low and high NPAR groups(n=359 each; **Table 1**), ensuring data representativeness and enabling better exploration of differences between patients with varying NPAR levels. Females constituted 77.4% of the cohort, with a higher proportion in the low-NPAR group (81.3% vs. 73.5%, P = 0.012). The low-NPAR group demonstrated a younger age distribution compared to the high-NPAR group (56 (48, 54) vs. 60 (51, 68), P<0.001) and had a lower percentage of patients aged 60˜79 (37.0% vs. 49.0%). Markers including RF, CRP, ESR, DDi, WBC, NEU, NEU percentage, PLT, DDi and NPAR, medication use like glucocorticoid, as well as the prevalence of OP, were significantly elevated in the high-NPAR group compared to the low-NPAR group (P<0.05,P<0.001).

**Table 1:** Comparison of baseline characteristics between RA patients in high and low NPAR groups.

Variables	Low-NPAR (n=359)	High-NPAR (n=359)	Overall (n=718)	P value
Demographics					
Gender, female, n(%)	292(81.3)	264(73.5)	556(77.4)	**0.012**	
Age, M (Q1,Q3)	56(48,54)	60(51,68)	58(50,66)	**<0.001**	
Age(years),n(%)				**0.007**	
≤39	43(12.0)	26(7.2)	69(9.6)		
40˜59	178(49.6)	152(42.3)	330(46.0)		
60˜79	133(37.0)	176(49.0)	309(43.0)		
≥80	5(1.4)	5(1.4)	10(1.4)		
Disease duration(years),n(%)				0.438	
<1	60(16.7)	73(20.3)	133(18.5)		
1˜5	110(30.6)	122(34.0)	232(32.3)		
6˜10	78(21.7)	70(19.5)	148(20.6)		
11˜20	77(21.4)	64(17.8)	141(19.6)	
>20	34(9.5)	30(8.4)	64(8.9)	
Smoking,n(%)	17(4.7)	41(11.4)	58(8.1)	**0.001**
Drinking,n(%)	7(1.9)	11(3.1)	18(2.5)	0.34
BMI(kg/m^2^),n(%)				0.148
<18.5	29(8.1)	42(11.7)	71(9.9)	
18.5˜24.9	225(62.7)	228(63.5)	453(63.1)	
25˜29.9	61(17.0)	45(12.5)	106(14.8)	
≥30	16(4.5)	10(2.8)	26(3.6)	
Missed	28(7.8)	34(9.5)	62(8.6)	
Comorbidities				
Diabetes,n(%)	51(14.2)	49(13.6)	100(13.9)	0.829
Hypertension,n(%)	88(24.5)	92(25.6)	180(25.1)	0.731
CVDs,n(%)	63(17.5)	74(17.8)	127(17.7)	0.922
Thyroid disease,n(%)	48(13.4)	30(8.4)	78(10.9)	**0.031**
Hyperuricemia/gout,n(%)	42(11.7)	24(6.7)	66(9.2)	**0.02**
Infectious diseases,n(%)	54(15.0)	78(21.7)	132(18.4)	**0.021**
ILD,n(%)	21(5.8)	27(7.5)	48(6.7)	0.37
Dyslipidemia,n(%)	228(63.5)	202(56.3)	430(59.9)	**0.048**
Osteoporosis,n(%)	95(26.5)	140(39.0)	235(32.7)	**<0.001**
Anemia,n(%)	106(34.1)	205(65.9)	311(43.3)	**<0.001**
Laboratory examination				
RF(IU/mL),n(%)				**<0.001**
≤20	102(28.4)	59(16.4)	161(22.4)	
>20	255(71.0)	295(82.2)	550(76.6)	
Unrecorded	2(0.6)	5(1.4)	7(1.0)	
ACPA(U/mL),n(%)				0.673
0˜5	55(15.3)	54(15.0)	109(15.2)	
5.1˜200	146(40.7)	148(41.2)	294(40.9)	
>200	130(36.2)	137(38.2)	267(37.2)	
Unrecorded	28(7.8)	20(5.6)	48(6.7)	
CRP(mg/L),n(%)				**<0.001**
0˜8	164(45.7)	28(7.8)	192(26.7)	
>8	194(54.0)	330(91.9)	524(73.0)	
Unrecorded	1(0.3)	1(0.3)	2(0.3)	
ESR(mm/h),n(%)				**<0.001**
Normal	96(26.7)	26(7.2)	122(17.0)	
Higher	257(71.6)	322(89.7)	579(80.6)	
Unrecorded	6(1.7)	11(3.1)	17(2.4)	
WBC(×10^9^/L), M (Q1,Q3)	5.91(4.74,7.44)	7.38(5.93,9.38)	6.62(5.28,8.29)	**<0.001**
RBC(×10^9^/L), M (Q1,Q3)	4.17(3.87,4.48)	3.87(3.54,4.27)	4.05(3.69,4.41)	**<0.001**
HGB(g/L), M (Q1,Q3)	119(110,129)	108(95,118)	114(101.75,124.25)	**<0.001**
NEU(×10^9^/L), M (Q1,Q3)	3.35(2.50,4.40)	5.21(4.07,6.97)	4.25(3.08,5.77)	**<0.001**
NEU percentage, Mean(SD)	56.64(9.02)	72.13(8.05)	64.39(11.54)	**<0.001**
LYM(×10^9^/L), M (Q1,Q3)	1.80(1.45,2.30)	1.36(1.06,1.75)	1.61(1.21,2.03)	**<0.001**
PLT(×10^9^/L), M (Q1,Q3)	274(227,341)	347(266,417)	309(238.75,386.25)	**<0.001**
DDi(mg/L), M (Q1,Q3)	0.95(0.38,2.36)	2.44(1.09,4.88)	1.56(0.67,3.59)	**<0.001**
TC(mmol/L), M (Q1,Q3)	4.68(3.92,5.32)	4.21(3.60,4.93)	4.39(3.74,5.14)	**<0.001**
TG(mmol/L), M (Q1,Q3)	1.07(0.77,1.46)	0.90(0.72,1.21)	1.0(0.74,1.36)	**<0.001**
HDL(mmol/L), M (Q1,Q3)	1.23(1.02,1.54)	1.15(0.94,1.41)	1.19(0.98,1.19)	**0.001**
LDL(mmol/L), M (Q1,Q3)	2.99(2.43,3.55)	2.69(2.19,3.21)	2.85(2.31,3.44)	**<0.001**
NPAR, M (Q1,Q3)	1.51(1.34,1.65)	2.03(1.88,2.24)	1.76(1.51,2.03)	**<0.001**
Medication Use				
NSAIDs, n(%)	236 (65.74)	242 (67.41)	478 (66.57)	0.635
GCs, n(%)	75 (20.89)	176 (49.03)	251 (34.96)	**<0.001**
MTX, n(%)	181 (50.42)	178 (49.58)	359 (50.00)	0.823
LEF, n(%)	66 (18.38)	55 (15.32)	121 (16.85)	0.273
HCQ, n(%)	31 (8.64)	31 (8.64)	62 (8.64)	1.000
SSZ, n(%)	1 (0.28)	3 (0.84)	4 (0.56)	0.616
IGU, n(%)	62 (17.27)	66 (18.38)	128 (17.83)	0.697
Tofacitinib, n(%)	82 (22.84)	73 (20.33)	155 (21.59)	0.414
Baritinib, n(%)	10 (2.79)	6 (1.67)	16 (2.23)	0.312
TNF-α inhibator, n(%)	40 (11.14)	52 (14.48)	92 (12.81)	0.180

SD, standard deviation; M, Median; Q_1_, 1st Quartile; Q_3_, 3rd Quartile.

The bolding was intended to denote statistical significance (p < 0.05).

Furthermore, compared to the low-NPAR group, the high-NPAR group exhibited a notably higher prevalence of smoking history and comorbidities such as infectious diseases and anemia (P<0.05), and showed significantly lower occurrences of thyroid disorders, dyslipidemia, and hyperuricemia/gout, as well as lower levels of LYM, RBC, HGB, TC, TG, HDL, and LDL (P<0.01). There were no significant differences between the groups in terms of disease duration, alcohol consumption history, BMI, ACPA levels, or the prevalence of diabetes mellitus, hypertension, CVDs, and ILD (all P>0.05).

### 3.2 Higher RA-OP prevalence in high-NPAR group

Partial variables in **Table 1** were subjected to univariate logistic regression analysis (**Supplementary Table S1**). Statistically significant variables identified were subsequently included in the multivariate analysis (**Table 2**). The findings indicate that elevated NPAR is more prevalent among RA patients who smoke, exhibit high levels of CRP and neutrophils, have OP, infectious diseases, and are of advanced age (P<0.001, P<0.05).

**Table 2:** Multivariate logistic regression analysis results of high NPAR and low NPAR groups.

Variables	*P*	OR (95%CI)	Variables	*P*	OR (95%CI)
Intercept	**0.002**	31.42 (3.40˜290.35)	Osteoporosis	**0.041**	1.82 (1.02˜3.24)
Age	**0.006**	1.03 (1.01˜1.05)	Hyperuricemia/gout	**0.008**	0.29 (0.11˜0.72)
Smoking	**0.039**	3.38 (1.06˜10.75)	Infectious disease	**0.022**	2.11 (1.11˜4.01)
CRP (mg/L)			WBC	**<0.001**	0.10 (0.06˜0.16)
0˜8		1.00 (Reference)	HGB	**<0.001**	0.94 (0.93˜0.96)
>8	**<0.001**	4.32 (2.28˜8.18)	NEU	**<0.001**	40.76 (21.19˜78.41)
Unrecorded	0.260	318.36 (0.01˜7281650.00)	TG	**0.009**	0.53 (0.33˜0.85)

The bolding was intended to denote statistical significance (p < 0.05).

### 3.3 NPAR independently associates with increased OP risk in RA patients

To determine whether NPAR is independently associated with OP risk, multi-model logistic regression analyses were conducted. As summarized in **Table 3**, elevated OP risk was observed when NPAR was modeled as a continuous variable in Models 1~3 and as a categorical variable in all four adjusted models. These findings suggest a consistent and positive association between NPAR and increased OP risk (P<0.05 for each).

**Table 3:** multi-model logistic regression analysis of the association between NPAR and RA-OP risk.

Variables	Model1		Model2		Model3		Model4	
	OR (95%CI)	*P*	OR (95%CI)	*P*	OR (95%CI)	*P*	OR (95%CI)	*P*
Higher	1.78 (1.29˜2.44)	**<0.001**	1.71 (1.19˜2.48)	**0.004**	1.77 (1.20˜2.61)	**0.004**	1.70 (1.01 ~ 2.88)	**0.049**
NPAR(continuous)	1.87 (1.30˜2.69)	**<0.001**	1.62 (1.07˜2.45)	**0.022**	1.70 (1.08˜2.66)	**0.021**	1.69 (0.71 ~ 4.04)	0.238

Model1: Crude.

Model2: Adjust: Age, Gender, Disease duration, BMI, Smoking and Drinking status.

Model3: Adjust: Age, Gender, Disease duration, BMI, Smoking and Drinking status; ILD, Anemia, Diabetes, Hypertension, Thyroid disease, Hyperuricemia/gout, Dyslipidemia, CVDs and Infectious diseases.

Model4: Adjust: Age, Gender, Disease duration, BMI, Smoking and Drinking status; ILD, Anemia, Diabetes, Hypertension, Thyroid disease, Hyperuricemia/gout, Dyslipidemia, CVDs and Infectious diseases; ACPA, RF, CRP, ESR, WBC, RBC, HGB, NEU, PLT, LYM, DDi, CHOL, TG, HDL, LDL; GCs.

The bolding was intended to denote statistical significance (p < 0.05).

In the initial unadjusted model (Model 1), a significant positive correlation was observed between continuous NPAR and OP risk (OR = 1.87, 95%CI:1.30˜2.69, P<0.001). Categorical analysis indicated that the high-NPAR group had approximately double the risk of the low-NPAR group (OR = 1.78, 95%CI:1.29˜2.44, P<0.001). Model 2 incorporated adjustments for demographic factors (age, gender), disease duration, smoking and alcohol history, and BMI. Model 3 further adjusted for comorbid conditions, and Model 4 expanded on Model 3 by including laboratory parameters and medication like GCs. In the fully adjusted Model 4, the high-NPAR group exhibited a 70% greater risk compared to the low-NPAR group (OR = 1.70, 95%CI:1.01 ~ 2.88, P = 0.049). These findings demonstrate that elevated NPAR levels, whether analyzed continuously or categorically, maintain consistent and independent associations with increased RA-OP risk across progressively stringent statistical adjustments.

### 3.4 Stratified analysis

**Figure 2:**
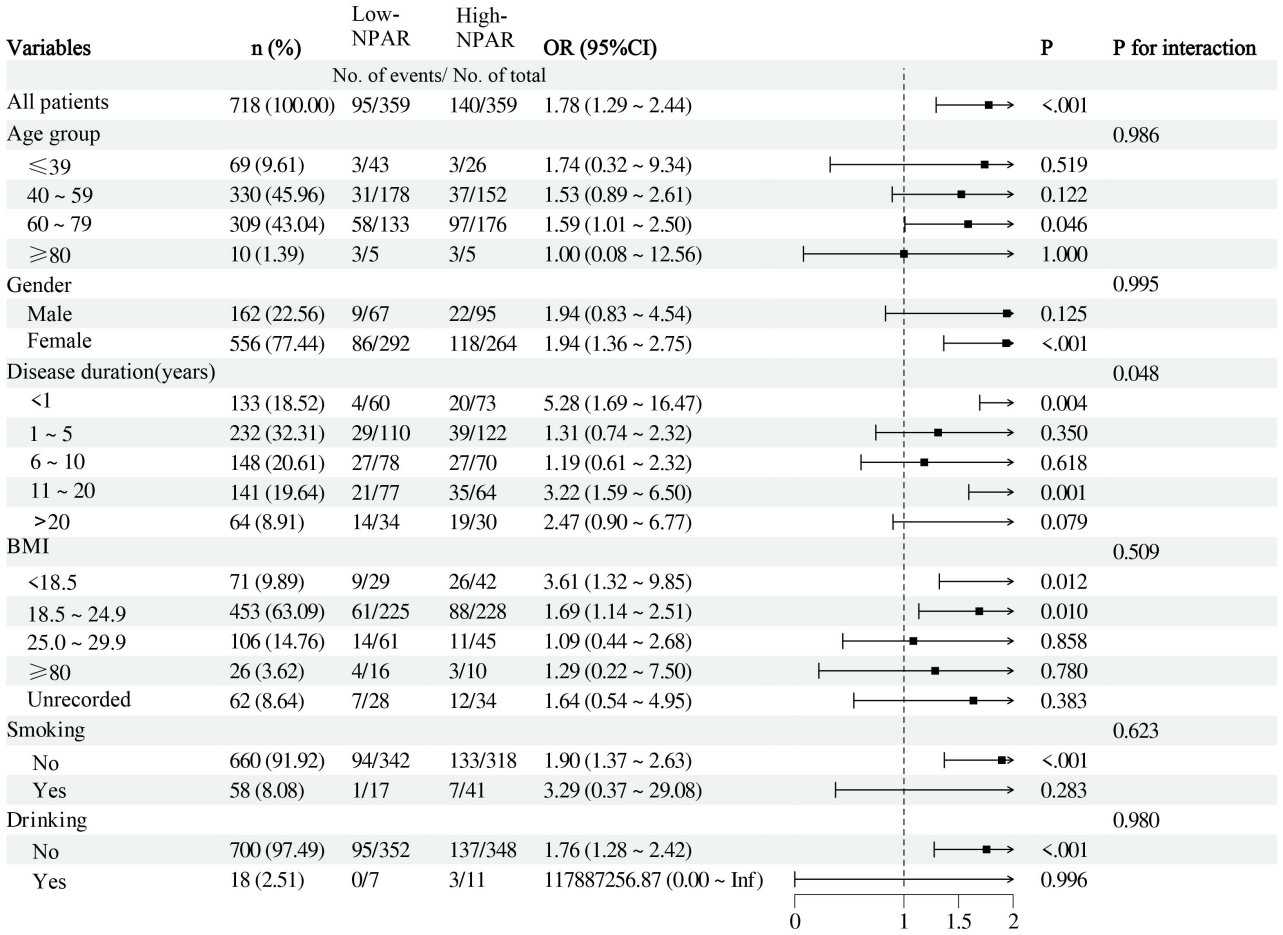
Subgroup Analysis of the Association between NPAR and RA-OP Risk.

We conducted subgroup analyses stratified by gender, age, disease duration, BMI, smoking, and drinking status, with NPAR as a continuous variable (**Figure 2**). No significant interactions were observed between NPAR and gender, age, smoking history, alcohol consumption history, or BMI (all P for interaction>0.05). However, a significant interaction was found between NPAR and disease duration concerning its effect on OP risk (P for interaction =0.048). Specifically, newly diagnosed patients with a disease duration of less than one year exhibited a substantially increased risk of OP with elevated NPAR (OR = 5.28, 95%CI: 1.69˜16.47, P = 0.004), while patients with a disease duration of 11˜20 years also demonstrated a significant elevation in OP risk (OR = 3.22, 95%CI:1.59˜6.50, P = 0.001). These results emphasize the importance of intensified monitoring for OP risk during the early and mid-term phases of RA. Further investigation is warranted to elucidate the impact of varying disease durations on the relationship between NPAR and RA-OP risk.

**Figure 3:**
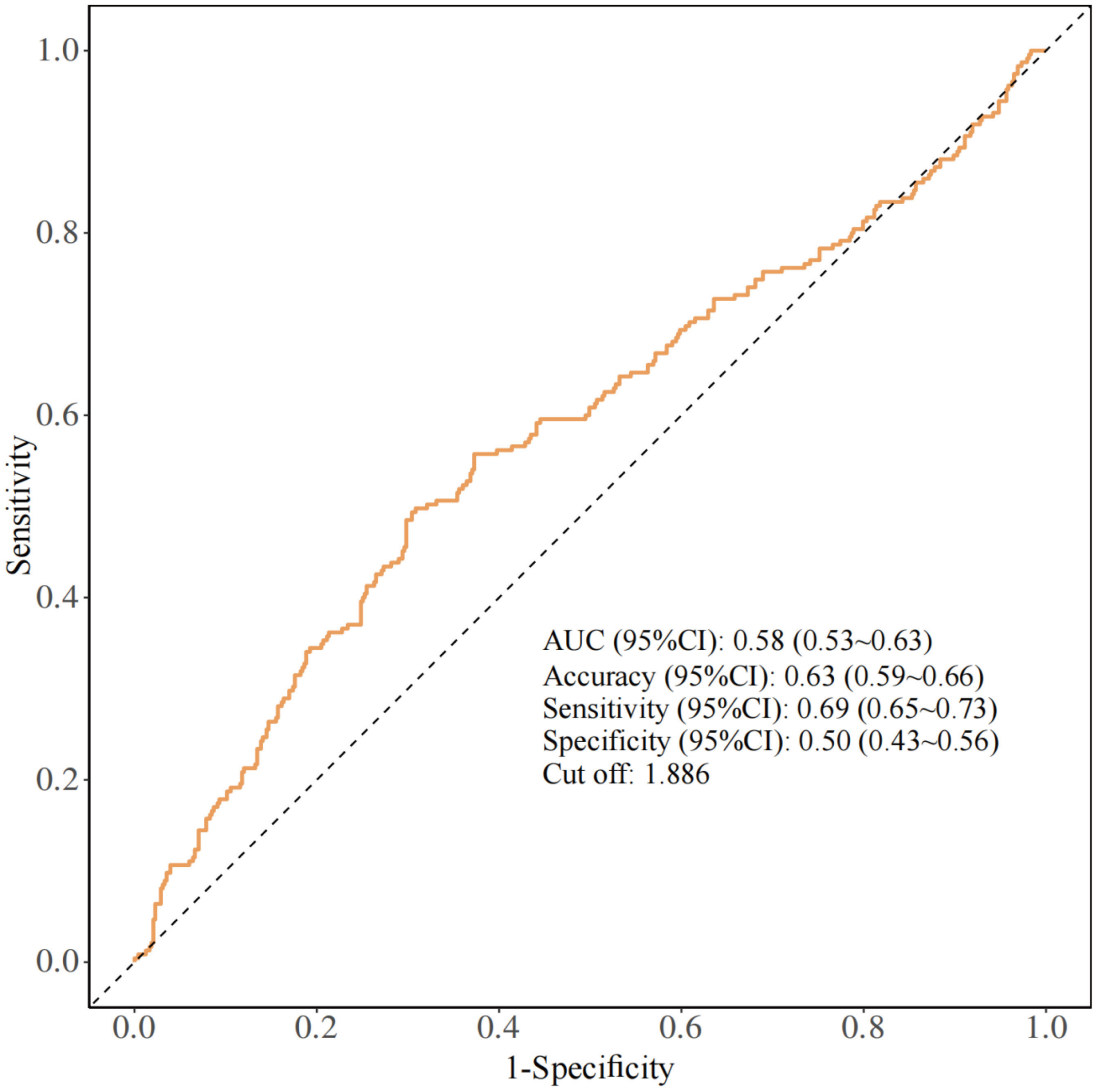
ROC curves of NPAR for predicting RA-OP risk.

The ROC curve was subsequently plotted based on the optimal Youden index to evaluate the predictive performance of NPAR for RA-OP risk. The analysis revealed an AUC of 0.58 (95% CI: 0.53~0.63), with a sensitivity of 69%, specificity of 50%, and an NPAR cut-off value of 1.886 (**Figure 3**).

### 3.5 Potential linear correlation between NPAR and RA-OP

**Figure 4:**
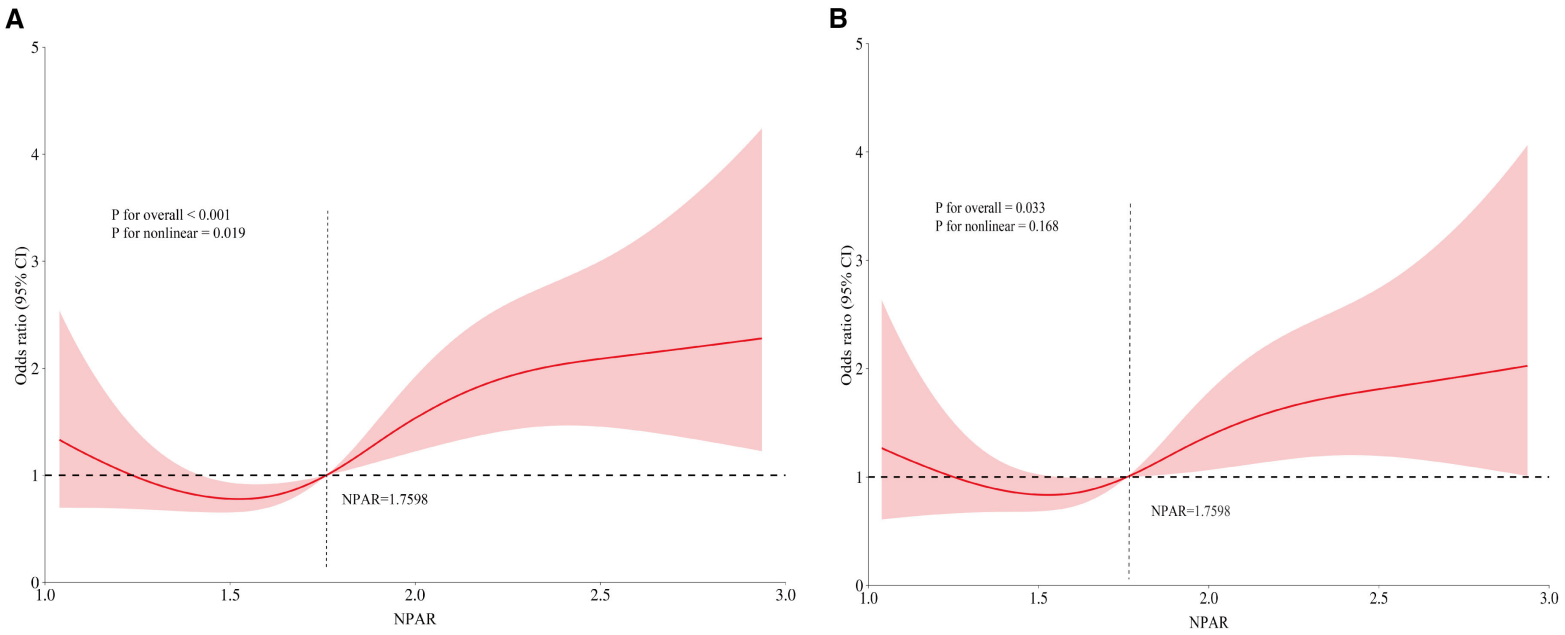
RCS illustrating the relationship between NPAR and RA-OP: **(A)** unadjusted RCS, **(B)** covariate-adjusted RCS.

The RCS curves, using NPAR median as a reference point, illustrated a J-shaped correlation between NPAR and RA-OP (**Figure 4**). Initially, prior to covariate adjustment, a statistically significant overall correlation was observed (P<0.001), along with indications of nonlinearity (P = 0.019) (**Figure 4A**). Subsequent adjustment for covariates including age, gender, disease duration, smoking and drinking history, and BMI revealed a persistently significant overall correlation (P = 0.033). However, the previously noted nonlinear relationship became insignificant (P = 0.168) (**Figure 4B**). These results suggest a potential linear relationship between this inflammatory biomarker and the incidence of OP in patients with RA.

Furthermore, utilizing data from the NHANES database (2005–2010, 2013–2014, 2017–2020), we assessed the correlation between NPAR and RA-OP in 1082 RA patients with femoral neck BMD measurements (**Supplementary Figure S1**). As shown in **Supplementary Table S2**, Serum NPAR levels were notably higher in RA patients with OP compared to those without OP (P = 0.01). Multivariate logistic regression results (**Supplementary Table S3**) revealed that female, older age, lower BMI, and a history of CVDs were independent risk factors for OP in RA. Subsequent multiple model logistic regression analysis showed that NPAR consistently exhibited a positive correlation with OP prevalence in RA patients in models 1-4 (Model1: OR = 1.15, 95%CI 1.04˜1.27, P = 0.009); Model2: OR = 1.15, 95%CI 1.02˜1.31, P = 0.033; Model3: OR = 1.14, 95%CI 0.98˜1.33, P = 0.089; Model4: OR = 1.33, 95%CI 1.04˜1.70, P = 0.027) (**Supplementary Table S4**). We constructed RCS plots to examine the relationship between NPAR and OP in RA patients (**Supplementary Figure S2**). The results showed a non-significant overall association before covariate adjustment (P = 0.085), with no significant evidence of nonlinearity (P = 0.602), suggesting a potential linear relationship between NPAR and RA-OP. This finding is consistent with the results of our retrospective clinical study. To optimize risk stratification and assess the consistency of NPAR’s impact on RA-OP incidence across different subgroups, we adjusted for covariates including age, gender, race/ethnicity, smoking status, alcohol consumption, BMI, PIR, education level, and marital status. Subgroup analyses were further performed based on comorbidities (hypertension, diabetes, dyslipidemia, cardiovascular diseases). The results demonstrated that each 1-unit increase in NPAR was associated with a significant 15% increase in OP risk among RA patients (P = 0.009). No significant interactions were observed between NPAR and any subgroup variables (P>0.05) (**Supplementary Figure S3**).

## 4 Discussion

RA is an autoimmune condition characterized by the activation of various immune cells and the release of inflammatory cytokines, leading to bone loss and structural damage. Prolonged glucocorticoid use and recurrent disease activity predispose RA patients to OP and fragility fractures. The NPAR, serving as an inflammatory biomarker, has been implicated in the pathogenesis of various autoimmune diseases. To explore the link between NPAR and OP in RA, we conducted an analysis of clinical data from the First Affiliated Hospital of Guangzhou University of Chinese Medicine. Our single-center study revealed that elevated NPAR is an independent predictor of OP risk in RA patients. This association remained significant even after adjusting for multiple covariates in logistic regression models. Nonlinear trend analyses demonstrated a positive linear correlation between NPAR levels and OP incidence. Validation using RA patient data from the NHANES database (2005-2020 cycles) further supported the robust association between NPAR and OP across multiracial populations. These results suggest that NPAR, a readily available and cost-effective biomarker, has clinical utility for stratifying OP risk and evaluating prognosis in RA patients.

NPAR has been extensively studied for its links to various health conditions, including depression (20), metabolic syndrome (21), cardiovascular disease mortality and all-cause mortality (22, 23), malignancies and their prognosis (24, 25), and infection risk (26). Research indicates that NPAR is significantly associated with autoimmune diseases, with a ten-unit rise in NPAR (adjusted for confounders) increasing the risk of psoriasis by 90% (OR = 1.90, 95% CI: 1.11˜3.26) (27), and it is a reliable predictor of intravenous immunoglobulin resistance in patients with Kawasaki disease (28). Recent investigations have highlighted NPAR as a critical predictor of mortality in patients with arthritis and those with arthritis-related hypertension, accurately predicting both all-cause and cardiovascular mortality (29, 30). Particularly, NPAR has been identified as an independent risk factor for heightened susceptibility to RA compared to osteoarthritis (14). Notably, there is currently a gap in research regarding the association between NPAR and OP.

OP arises from a disruption in the finely tuned physiological process of “bone remodeling”. Inflammatory conditions are commonly linked to OP, with the immune system intricately intertwined with bone health and disease (31). Neutrophils play a crucial role in maintaining bone balance but become hyperactive in the absence of estrogen. This hyperactivity results in elevated osteoclast production through the release of reactive oxygen species and expression of RANKL, ultimately triggering osteoblast apoptosis and fostering postmenopausal OP (32). Research indicates that neutrophils expressing RANKL in inflammatory disorders are associated with decreased BMD. In RA patients, synovial neutrophils exhibit both membrane-bound RANKL (mRANKL) and RANK while secreting osteoprotegerin, highlighting their dual impact on bone remodeling in RA (31). Neutrophils are implicated in the computation of various inflammatory markers, such as the SII (calculated as (neutrophil count×platelet count)/lymphocyte count). Elevated SII and other neutrophil-related markers like NLR and the platelet count multiplied by the neutrophil count (PPN) are significantly linked to reduced BMD and heightened risk of OP (33).

Low serum albumin (ALB) is independently associated with OP development and may represent a risk factor in postmenopausal RA patients (34). Similarly, low albumin levels in male patients with type 2 diabetes are a risk factor for OP (35). OP patients have significantly lower serum albumin levels compared to individuals with normal or low BMD. Decreased serum albumin levels are strongly associated with an elevated risk of osteoporotic fractures (OR = 0.073; 95%CI:0.045˜0.119) (36). Previous studies have demonstrated that OP in various anatomical sites such as the femoral neck, total femur, and lumbar vertebrae is independently linked to hypoalbuminemia (9). Serum ALB, as a marker of nutritional status, has attracted considerable attention due to its associations with OP related to inflammatory and metabolic disorders. Thyroid disorders are known to hinder albumin synthesis, promote bone resorption and impede bone repair, leading to reduced bone mass and increased bone fragility. In our analysis comparing RA patients with and without OP (**Supplementary Figure S3**), the prevalence of thyroid disease did not differ significantly between the groups (P = 0.375), suggesting that its association with RA-OP may be influenced by other factors warranting further exploration. Overall, this study identifies low serum albumin levels as an independent predictor of increased OP risk in RA. The notable predictive capacity and straightforward computation of NPAR make it a promising novel inflammatory marker deserving of further investigation.

In assessing the predictive capability of NPAR for RA-OP risk using NHANES 2005-2020 data, we noted a potential elevation in OP risk among RA patients with concurrent CVDs (**Supplementary Table S2**). Individuals with RA exhibit a twofold higher risk of CVDs compared to the general population, with CVDs emerging as a predominant cause of mortality in this cohort (37). CVDs, recognized as chronic inflammatory conditions, are marked by notably heightened levels of inflammatory cytokines like IL-6, TNF-α, and IL-1β (38).Importantly, therapies targeting inflammation such as TNF-α and IL-6 inhibitors have shown promise in reducing CVDs incidence in RA (39). Shared risk factors and underlying mechanisms including inflammation, oxidative stress, aging, and sedentary lifestyle contribute to both OP and CVDs (40), potentially elucidating the observed association between CVDs and OP in RA. Nevertheless, our examination of the consistency of NPAR’s impact on RA-OP risk across different comorbidities revealed no significant NPAR-CVDs interaction effect (**Supplementary Figure S3**), indicating that NPAR’s influence on RA-OP risk is independent of CVDs status.

Several limitations exist in this study. Firstly, both surveys were cross-sectional and observational studies, which precluded causal inference. Secondly, despite comprehensive adjustment for covariates, potential confounders not captured, such as vitamin D, physical activity and cumulative glucocorticoid dose, may potentially affect the results. Thirdly, the single-timepoint measurement of NPAR is inadequate to understand its dynamic changes over time or after interventions. Fourthly, the use of inpatient data only and the limited sample size in clinical research may restrict the generalizability of the findings. Finally, NPAR can be affected by acute infection, stress, and liver function, while albumin may decrease due to non-nutritional factors such as inflammation or fluid overload. Moreover, the cross-sectional design impedes establishing a causal relationship between NPAR and RA-OP. Therefore, future research necessitates larger-scale, multicenter, and prospective studies to corroborate these findings and investigate strategies for augmenting the predictive capacity of NPAR for RA-OP risk in conjunction with other biomarkers.

## 5 Conclusion

Drawing on data from a single-center cohort in China and a nationally representative sample in the United States, this research demonstrates that increased NPAR is a standalone predictor of OP risk in adult RA patients. These results offer strong support for utilizing NPAR as a biomarker for risk assessment and prognosis, bridging the existing gap in understanding the relationship between NPAR and OP in RA. The study underscores the practical value of NPAR as a readily available and cost-effective biomarker. Integrating NPAR assessment into standard clinical practice can enhance risk assessment and preventive strategies for OP in individuals with RA.

## Data Availability

The original contributions presented in the study are included in the article/**Supplementary Material**. Further inquiries can be directed to the corresponding authors.
