# Supplementary figures and images for: Neutrophil-to-albumin ratio: a novel predictor of osteoporosis in rheumatoid arthritis
Source: Front Immunol. 2025 Sep 17;16:1666884. doi: 10.3389/fimmu.2025.1666884 (PMC12484169; doi:10.3389/fimmu.2025.1666884)

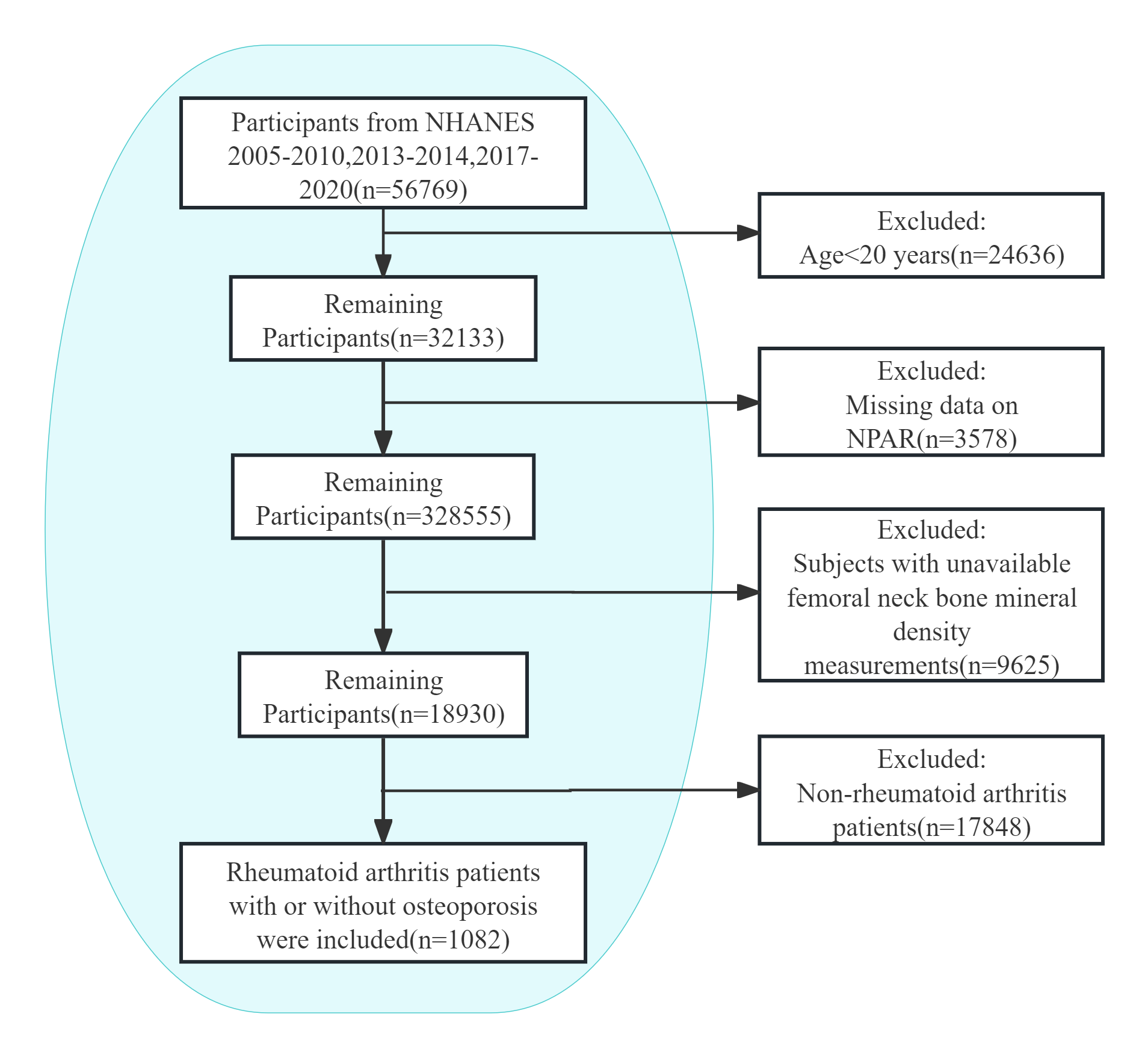

Supplement: Supplementary file 1 [file Image1.tif]

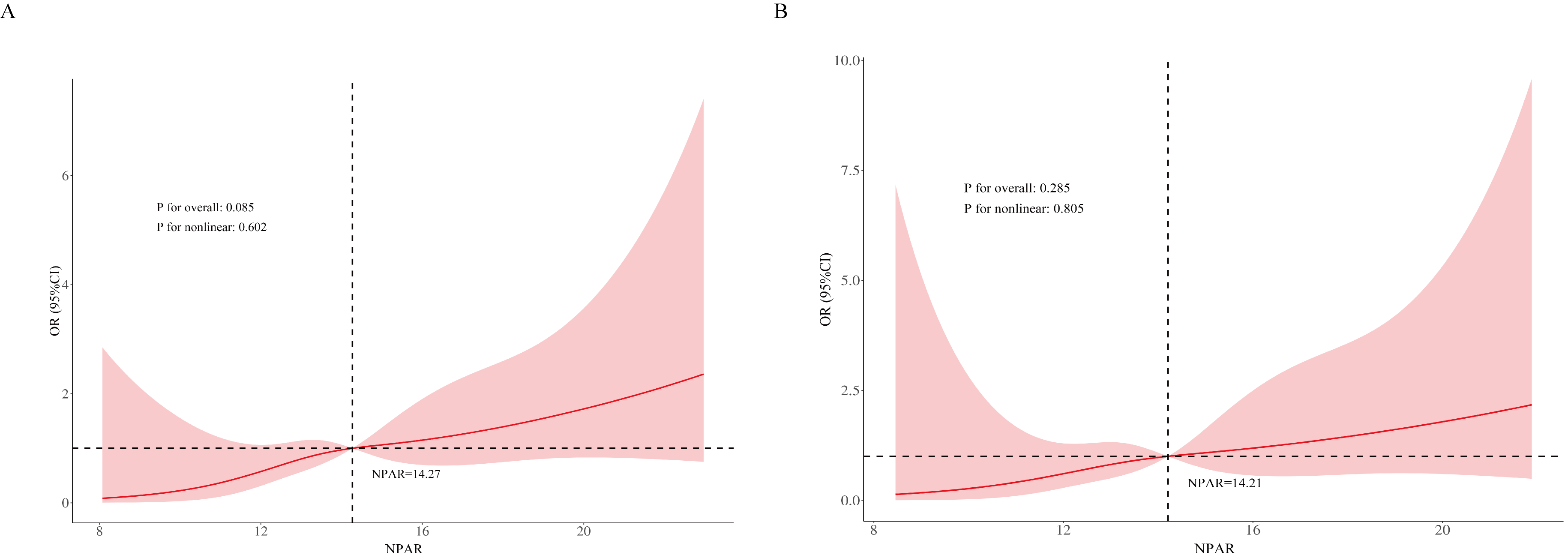

Supplement: Supplementary file 2 [file Image2.tif]

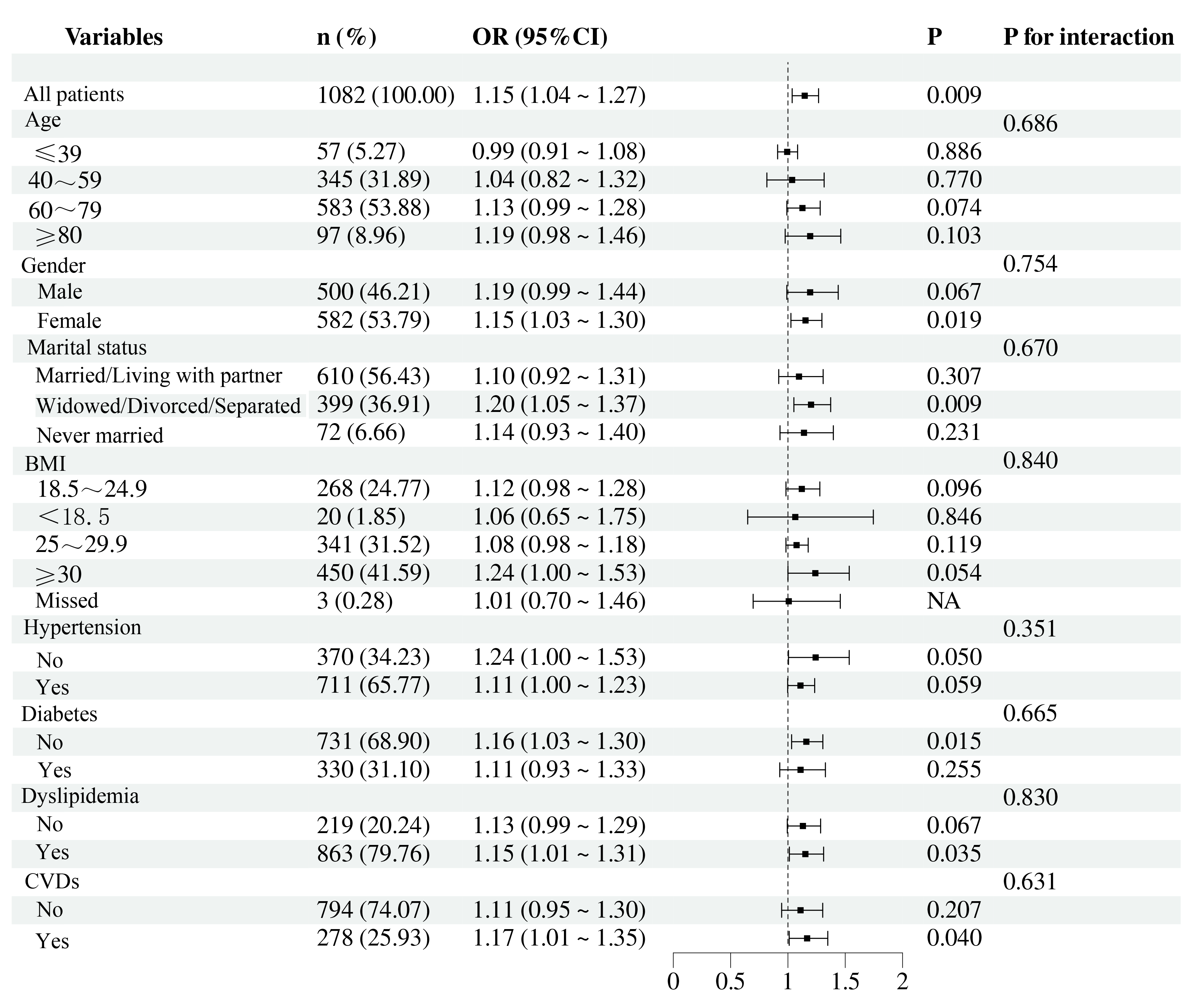

Supplement: Supplementary file 3 [file Image3.tif]
