# Supplementary material for: Neutrophil-to-albumin ratio: a novel predictor of osteoporosis in rheumatoid arthritis
Source: Front Immunol. 2025 Sep 17;16:1666884. doi: 10.3389/fimmu.2025.1666884 (PMC12484169; doi:10.3389/fimmu.2025.1666884)
Supplement: Supplementary file 4 [file Table1.docx]

**Table S1 Factors Associated with NPAR: Results of Univariate and Multivariate Logistic Regression Analyses**

### Table1 Univariate Logistic Regression Analyses for Factors Associated with NPAR

| Variables | β | S.E | Z | *P* | OR (95%CI) | Variables | β | S.E | Z | *P* | OR (95%CI) |
| --- | --- | --- | --- | --- | --- | --- | --- | --- | --- | --- | --- |
| Age | 0.02 | 0.01 | 3.95 | **<0.001** | 1.02 (1.01 ~ 1.04) | Dyslipidemia |  |  |  |  |  |
| Gender |  |  |  |  |  | No |  |  |  |  | 1.00 (Reference) |
| Male |  |  |  |  | 1.00 (Reference) | Yes | -0.30 | 0.15 | -1.98 | **0.048** | 0.74 (0.55 ~ 0.99) |
| Female | -0.45 | 0.18 | -2.49 | **0.013** | 0.64 (0.45 ~ 0.91) | CVDs |  |  |  |  |  |
| Disease duration |  |  |  |  |  | No |  |  |  |  | 1.00 (Reference) |
| ＜1 |  |  |  |  | 1.00 (Reference) | Yes | 0.02 | 0.20 | 0.10 | 0.922 | 1.02 (0.69 ~ 1.50) |
| 1～5 | -0.09 | 0.22 | -0.42 | 0.672 | 0.91 (0.59 ~ 1.40) | Infectious diseases |  |  |  |  |  |
| 6～10 | -0.30 | 0.24 | -1.27 | 0.204 | 0.74 (0.46 ~ 1.18) | No |  |  |  |  | 1.00 (Reference) |
| 11～20 | -0.38 | 0.24 | -1.57 | 0.117 | 0.68 (0.42 ~ 1.10) | Yes | 0.45 | 0.20 | 2.30 | **0.021** | 1.57 (1.07 ~ 2.30) |
| ＞20 | -0.32 | 0.31 | -1.05 | 0.292 | 0.73 (0.40 ~ 1.32) | Osteoporosis |  |  |  |  |  |
| BMI |  |  |  |  |  | No |  |  |  |  | 1.00 (Reference) |
| ＜18.5 |  |  |  |  | 1.00 (Reference) | Yes | 0.57 | 0.16 | 3.56 | **<0.001** | 1.78 (1.29 ~ 2.44) |
| 18.5～24.9 | -0.36 | 0.26 | -1.38 | 0.168 | 0.70 (0.42 ~ 1.16) | RF |  |  |  |  |  |
| 25～29.9 | -0.67 | 0.31 | -2.17 | **0.030** | 0.51 (0.28 ~ 0.94) | ≤20 |  |  |  |  | 1.00 (Reference) |
| ≥30 | -0.84 | 0.47 | -1.79 | 0.074 | 0.43 (0.17 ~ 1.08) | ＞20 | 0.69 | 0.18 | 3.76 | **<0.001** | 2.00 (1.39 ~ 2.87) |
| Missed | -0.18 | 0.35 | -0.50 | 0.616 | 0.84 (0.42 ~ 1.67) | Unrecorded | 1.46 | 0.85 | 1.72 | 0.086 | 4.32 (0.81 ~ 22.98) |
| Smoking |  |  |  |  |  | ACPA |  |  |  |  |  |
| No |  |  |  |  | 1.00 (Reference) | 0～5 |  |  |  |  | 1.00 (Reference) |
| Yes | 0.95 | 0.30 | 3.19 | **0.001** | 2.59 (1.44 ~ 4.66) | 5.1～200 | 0.03 | 0.22 | 0.14 | 0.887 | 1.03 (0.67 ~ 1.60) |
| Drinking |  |  |  |  |  | ＞200 | 0.07 | 0.23 | 0.31 | 0.756 | 1.07 (0.69 ~ 1.68) |
| No |  |  |  |  | 1.00 (Reference) | Unrecorded | -0.32 | 0.35 | -0.91 | 0.363 | 0.73 (0.37 ~ 1.44) |
| Yes | 0.46 | 0.49 | 0.95 | 0.344 | 1.59 (0.61 ~ 4.15) | CRP |  |  |  |  |  |
| ILD |  |  |  |  |  | 0～8 |  |  |  |  | 1.00 (Reference) |
| No |  |  |  |  | 1.00 (Reference) | ＞8 | 2.30 | 0.22 | 10.28 | **<0.001** | 9.96 (6.43 ~ 15.44) |
| Yes | 0.27 | 0.30 | 0.89 | 0.371 | 1.31 (0.73 ~ 2.36) | Unrecorded | 1.77 | 1.43 | 1.24 | 0.216 | 5.86 (0.36 ~ 96.38) |
| Anemia |  |  |  |  |  | ESR group |  |  |  |  |  |
| No |  |  |  |  | 1.00 (Reference) | Normal |  |  |  |  | 1.00 (Reference) |
| Yes | 1.16 | 0.16 | 7.35 | **<0.001** | 3.18 (2.33 ~ 4.32) | Higher | 1.53 | 0.24 | 6.48 | **<0.001** | 4.63 (2.91 ~ 7.35) |
| Diabetes |  |  |  |  |  | Unrecorded | 1.91 | 0.55 | 3.45 | **<0.001** | 6.77 (2.29 ~ 20.03) |
| No |  |  |  |  | 1.00 (Reference) | WBC | 0.31 | 0.04 | 8.36 | **<0.001** | 1.37 (1.27 ~ 1.47) |
| Yes | -0.05 | 0.22 | -0.22 | 0.829 | 0.95 (0.63 ~ 1.46) | RBC | -0.94 | 0.15 | -6.40 | **<0.001** | 0.39 (0.29 ~ 0.52) |
| Hypertension |  |  |  |  |  | HGB | -0.04 | 0.00 | -8.02 | **<0.001** | 0.96 (0.95 ~ 0.97) |
| No |  |  |  |  | 1.00 (Reference) | NEU | 0.69 | 0.06 | 11.46 | **<0.001** | 1.99 (1.77 ~ 2.24) |

**Table1 Continued**

| Variables | β | S.E | Z | *P* | OR (95%CI) | Variables | β | S.E | Z | *P* | OR (95%CI) |
| --- | --- | --- | --- | --- | --- | --- | --- | --- | --- | --- | --- |
| Yes | 0.06 | 0.17 | 0.34 | 0.731 | 1.06 (0.76 ~ 1.49) | PLT | 0.01 | 0.00 | 7.39 | **<0.001** | 1.01 (1.01 ~ 1.01) |
| Thyroid disease |  |  |  |  |  | LYM | -0.92 | 0.13 | -7.06 | **<0.001** | 0.40 (0.31 ~ 0.51) |
| No |  |  |  |  | 1.00 (Reference) | DDi | 0.20 | 0.03 | 6.20 | **<0.001** | 1.22 (1.14 ~ 1.30) |
| Yes | -0.53 | 0.25 | -2.14 | **0.032** | 0.59 (0.36 ~ 0.96) | CHOL | -0.37 | 0.08 | -4.78 | **<0.001** | 0.69 (0.60 ~ 0.81) |
| Hyperuricemia/gout |  |  |  |  |  | TG | -0.59 | 0.14 | -4.21 | **<0.001** | 0.55 (0.42 ~ 0.73) |
| No |  |  |  |  | 1.00 (Reference) | HDL | -0.65 | 0.20 | -3.21 | **0.001** | 0.52 (0.35 ~ 0.78) |
| Yes | -0.61 | 0.27 | -2.30 | **0.022** | 0.54 (0.32 ~ 0.91) | LDL | -0.32 | 0.09 | -3.70 | **<0.001** | 0.73 (0.61 ~ 0.86) |

### Table2 Multivariate logistic regression results

| Variables | β | S.E | Z | *P* | OR (95%CI) |
| --- | --- | --- | --- | --- | --- |
|  |  |  |  |  |  |
| Intercept | 3.45 | 1.13 | 3.04 | **0.002** | 31.42 (3.40 ~ 290.35) |
| Age | 0.03 | 0.01 | 2.76 | **0.006** | 1.03 (1.01 ~ 1.05) |
| Smoking |  |  |  |  |  |
| No |  |  |  |  | 1.00 (Reference) |
| Yes | 1.22 | 0.59 | 2.06 | **0.039** | 3.38 (1.06 ~ 10.75) |
| Hyperuricemia/gout |  |  |  |  |  |
| No |  |  |  |  | 1.00 (Reference) |
| Yes | -1.25 | 0.47 | -2.65 | **0.008** | 0.29 (0.11 ~ 0.72) |
| Infectious diseases |  |  |  |  |  |
| No |  |  |  |  | 1.00 (Reference) |
| Yes | 0.75 | 0.33 | 2.29 | **0.022** | 2.11 (1.11 ~ 4.01) |
| Osteoporosis |  |  |  |  |  |
| No |  |  |  |  | 1.00 (Reference) |
| Yes | 0.60 | 0.29 | 2.04 | **0.041** | 1.82 (1.02 ~ 3.24) |
| CRP |  |  |  |  |  |
| 0～8 |  |  |  |  | 1.00 (Reference) |
| ＞8 | 1.46 | 0.33 | 4.49 | **<0.001** | 4.32 (2.28 ~ 8.18) |
| Unrecorded | 5.76 | 5.12 | 1.13 | 0.260 | 318.36 (0.01 ~ 7281650.00) |
| WBC | -2.31 | 0.24 | -9.68 | **<0.001** | 0.10 (0.06 ~ 0.16) |
| HGB | -0.06 | 0.01 | -6.91 | **<0.001** | 0.94 (0.93 ~ 0.96) |
| NEU | 3.71 | 0.33 | 11.11 | **<0.001** | 40.76 (21.19 ~ 78.41) |
| TG | -0.64 | 0.24 | -2.63 | **0.009** | 0.53 (0.33 ~ 0.85) |
| OR: Odds Ratio, CI: Confidence Interval | | | | | |
