# Supplementary material for: Neutrophil-to-albumin ratio: a novel predictor of osteoporosis in rheumatoid arthritis
Source: Front Immunol. 2025 Sep 17;16:1666884. doi: 10.3389/fimmu.2025.1666884 (PMC12484169; doi:10.3389/fimmu.2025.1666884)
Supplement: Supplementary file 5 [file Table2.doc]

TABLE S2 Comparison of Baseline Characteristics in RA Patients With Versus Without OP

| Variable | RA without OP (n=1030) | RA with OP(n=52) | Total (n = 1082) | *P* |
| --- | --- | --- | --- | --- |
| **Demographics** |  |  |  |  |
| Age, M (Q1,Q3) | 62.0 (53.0, 70.0) | 73.0 (65.0, 79.0) | 62.0 (53.0, 70.0) | **＜0.001** |
| Gender, Female, n(%) | 540 (54.95) | 42 (90.64) | 582 (56.22) | **＜0.001** |
| BMI(kg/m^2^), n(%) |  |  |  | **＜0.001** |
| ＜18.5 | 14 (1.07) | 6 (25.45) | 20 (1.95) |  |
| 18.5～24.9 | 238 (23.85) | 30 (48.44) | 268 (24.73) |  |
| 25～29.9 | 331 (30.81) | 10 (18.07) | 341 (30.36) |  |
| ≥30 | 444 (43.95) | 6 (8.05) | 450 (42.67) |  |
| Missed | 3 (0.31) | 0 (0.00) | 3 (0.30) |  |
| Race, n(%) |  |  |  | 0.118 |
| Mexican American | 145 (13.35) | 9 (11.53) | 154 (13.29) |  |
| Other Hispanic | 87 (8.91) | 7 (12.81) | 94 (9.05) |  |
| Non-Hispanic White | 434 (44.40) | 24 (37.77) | 458 (44.16) |  |
| Non-Hispanic Black | 308 (29.24) | 7 (22.81) | 315 (29.01) |  |
| Other Race | 56 (4.10) | 5 (15.09) | 61 (4.49) |  |
| Marital status, n(%) |  |  |  | 0.054 |
| Married/Living with partner | 592 (57.48) | 18 (31.74) | 610 (56.55) |  |
| Widowed/Divorced/Separated | 367 (35.32) | 32 (58.42) | 399 (36.15) |  |
| Never married | 70 (7.20) | 2 (9.84) | 72 (7.29) |  |
| Educational level, n(%) |  |  |  | 0.352 |
| Less than 9th grade | 154 (8.96) | 5 (10.15) | 159 (9.00) |  |
| 9～11th grade | 172 (12.72) | 12 (24.90) | 184 (13.15) |  |
| High school graduate | 257 (29.11) | 15 (31.60) | 272 (29.20) |  |
| Some college or associates degree | 314 (33.56) | 14 (18.18) | 328 (33.01) |  |
| College graduate or above | 132 (15.65) | 6 (15.17) | 138 (15.63) |  |
| PIR, n(%) |  |  |  | 0.461 |
| ≤1.3 | 349 (38.47) | 25 (48.89) | 374 (38.85) |  |
| 1.3～3.5 | 346 (35.57) | 12 (34.45) | 358 (35.53) |  |
| ≥3.5 | 244 (25.96) | 9 (16.67) | 253 (25.62) |  |
| Smoking Status, n(%) |  |  |  | **0.006** |
| Never | 446 (40.38) | 32 (57.40) | 478 (40.99) |  |
| Former | 334 (33.28) | 8 (6.90) | 342 (32.34) |  |
| Now | 249 (26.25) | 12 (35.70) | 261 (26.59) |  |

**TABLE S2** Continued

| Variable | RA without OP (n=1030) | RA with OP(n=52) | Total (n = 1082) | *P* |
| --- | --- | --- | --- | --- |
| Missed | 1 (0.09) | 0 (0.00) | 1 (0.09) |  |
| Drinking Status, n(%) |  |  |  | 0.086 |
| No | 225 (20.71) | 16 (38.23) | 241 (21.34) |  |
| Yes | 497 (45.78) | 18 (37.75) | 515 (45.50) |  |
| Missed | 308 (33.50) | 18 (24.02) | 326 (33.16) |  |
| **Comorbidities** | | | | |
| Hypertension, n(%) | 672 (64.01) | 39 (75.17) | 711 (64.41) | 0.294 |
| Diabetes, n(%) | 318 (31.08) | 12 (15.57) | 330 (30.52) | **0.028** |
| Dyslipidemia, n(%) | 822 (81.08) | 41 (80.98) | 863 (81.08) | 0.989 |
| CVDs, n(%) | 256 (25.50) | 22 (45.47) | 278 (26.19) | **0.029** |
| **Laboratory Examination** | | | | |
| CRP, M (Q1,Q3) | 2.50 (1.31, 4.83) | 2.60 (1.48, 4.80) | 2.53 (1.32, 4.83) | 0.568 |
| ALB, M (Q1,Q3) | 41.00 (39.00, 43.00) | 42.00 (40.00, 44.00) | 41.00 (39.00, 43.00) | 0.226 |
| WBC, M (Q1,Q3) | 6.90 (5.70, 8.50) | 7.10 (5.90, 8.60) | 6.90 (5.70, 8.60) | 0.596 |
| LYM, M (Q1,Q3) | 2.00 (1.50, 2.50) | 2.00 (1.50, 2.30) | 2.00 (1.50, 2.50) | 0.560 |
| NEU, M (Q1,Q3) | 4.10 (3.10, 5.30) | 4.40 (3.00, 5.20) | 4.10 (3.10, 5.30) | 0.656 |
| RBC, M (Q1,Q3) | 4.61 (4.26, 4.87) | 4.48 (4.28, 4.87) | 4.60 (4.26, 4.87) | 0.737 |
| HGB, Mean (SE) | 14.07 (0.06) | 13.88 (0.30) | 14.06 (0.06) | 0.524 |
| PLT, M (Q1,Q3) | 242.00 (204.00, 291.00) | 246.00 (237.00, 330.00) | 242.00 (205.00, 291.00) | **0.025** |
| NPAR, Mean (SE) | 14.39 (0.17) | 15.69 (0.48) | 14.43 (0.16) | **0.010** |
| NPAR, n(%) |  |  |  | **0.025** |
| Q1 | 277 (25.53) | 7 (7.38) | 284 (24.88) |  |
| Q2 | 258 (24.43) | 17 (34.58) | 275 (24.79) |  |
| Q3 | 249 (25.49) | 9 (17.30) | 258 (25.20) |  |
| Q4 | 246 (24.55) | 19 (40.75) | 265 (25.13) |  |
