# Supplementary material for: Neutrophil-to-albumin ratio: a novel predictor of osteoporosis in rheumatoid arthritis
Source: Front Immunol. 2025 Sep 17;16:1666884. doi: 10.3389/fimmu.2025.1666884 (PMC12484169; doi:10.3389/fimmu.2025.1666884)
Supplement: Supplementary file 6 [file Table3.doc]

TABLE S3 Multivariate Logistic Regression Analysis of Risk Factors Associated with OP in RA

| Variables | *P* | OR (95%CI) |  | Variables | *P* | OR (95%CI) |
| --- | --- | --- | --- | --- | --- | --- |
| Intercept | **＜0.001** | 0.00 (0.00～0.00) |  | BMI(kg/m^2^) |  |  |
| Age | **＜0.001** | 1.15 (1.09～1.22) |  | 18.5～24.9 |  | 1.00 (Reference) |
| Gender,Female | **＜0.001** | 7.66 (2.50～23.45) |  | ＜18.5 | **0.002** | 17.38 (3.13～96.55) |
| Marital status |  |  |  | 25～29.9 | **0.001** | 0.29 (0.14～0.58) |
| Married/Living with partner |  | 1.00 (Reference) |  | ≥30 | **0.001** | 0.11 (0.03～0.40) |
| Widowed/Divorced/Separated | 0.682 | 1.25 (0.43～3.68) |  | Missed | **＜0.001** | 0.00 (0.00～0.00) |
| Never married | **0.010** | 14.44 (1.98～105.29) |  | CVDs | **0.012** | 3.11 (1.31～7.36) |
| Smoking Status |  |  |  | NPAR |  |  |
| Never |  | 1.00 (Reference) |  | Q1 |  | 1.00 (Reference) |
| Former | **0.009** | 0.18 (0.05～0.62) |  | Q2 | **0.002** | 5.68 (2.00～16.12) |
| Now | 0.985 | 1.01 (0.34～3.01) |  | Q3 | 0.271 | 2.12 (0.56～8.03) |
| Missed | **＜0.001** | 0.00 (0.00～0.00) |  | Q4 | **0.015** | 5.32 (1.43～19.76) |
