# Supplementary material for: Neutrophil-to-albumin ratio: a novel predictor of osteoporosis in rheumatoid arthritis
Source: Front Immunol. 2025 Sep 17;16:1666884. doi: 10.3389/fimmu.2025.1666884 (PMC12484169; doi:10.3389/fimmu.2025.1666884)
Supplement: Supplementary file 7 [file Table4.doc]

TABLE S4 Multi-model logistic regression analysis of the association between NPAR and RA-OP risk

| Variables | Model1 | | Model2 | | Model3 | | Model4 | |
| --- | --- | --- | --- | --- | --- | --- | --- | --- |
|  | OR (95%CI) | *P* | OR (95%CI) | *P* | OR (95%CI) | *P* | OR (95%CI) | *P* |
| NPAR |  |  |  |  |  |  |  |  |
| Q1 | 1.00 (Reference) |  | 1.00 (Reference) |  | 1.00 (Reference) |  | 1.00 (Reference) |  |
| Q2 | 4.90 (1.62～14.78) | **0.006** | 7.56 (2.44～23.38) | **＜0.001** | 8.08 (1.84～35.35) | **0.008** | 16.38 (2.89～92.76) | **0.003** |
| Q3 | 2.35 (0.68～8.11) | 0.181 | 1.62 (0.36～7.35) | 0.537 | 1.90 (0.39～9.15) | 0.430 | 1.49 (0.29～7.69) | 0.638 |
| Q4 | 5.74 (1.95～16.90) | **0.002** | 9.65 (2.50～37.28) | **0.002** | 10.52 (2.05～53.86) | **0.007** | 25.60 (3.20～204.77) | **0.004** |
| NPAR(continuous) | 1.15 (1.04～1.27) | **0.009** | 1.15 (1.02～1.31) | **0.033** | 1.14 (0.98～1.33) | 0.089 | 1.33 (1.04～1.70) | **0.027** |
