# Supplementary material for: Neutrophil-to-albumin ratio: a novel predictor of osteoporosis in rheumatoid arthritis
Source: Front Immunol. 2025 Sep 17;16:1666884. doi: 10.3389/fimmu.2025.1666884 (PMC12484169; doi:10.3389/fimmu.2025.1666884)
Supplement: Supplementary file 8 [file Table5.docx]

### Table S5 Comparison of Baseline Characteristics Between RA Patients With and Without Osteoporosis

| Variables | Total (n = 718) | RA without osteoporosis (n = 483) | RA with osteoporosis (n = 235) | Statistic | *P* |
| --- | --- | --- | --- | --- | --- |
|  |  |  |  |  |  |
| **Demographics** |  |  |  |  |  |
| Age, M (Q₁, Q₃) | 58.00 (50.00, 66.00) | 54.00 (46.00, 63.00) | 64.00 (58.00, 70.00) | Z=-10.48 | **<0.001** |
| Age(years),n(%) |  |  |  | χ²=86.16 | **<0.001** |
| ≤39 | 69 (9.61) | 63 (13.04) | 6 (2.55) |  |  |
| 40～59 | 330 (45.96) | 262 (54.24) | 68 (28.94) |  |  |
| 60～79 | 309 (43.04) | 154 (31.88) | 155 (65.96) |  |  |
| ≥80 | 10 (1.39) | 4 (0.83) | 6 (2.55) |  |  |
| Gender, n(%) |  |  |  | χ²=17.56 | **<0.001** |
| Male | 162 (22.56) | 131 (27.12) | 31 (13.19) |  |  |
| Female | 556 (77.44) | 352 (72.88) | 204 (86.81) |  |  |
| Disease duration(years),n(%) |  |  |  | χ²=28.64 | **<0.001** |
| ＜1 | 133 (18.52) | 109 (22.57) | 24 (10.21) |  |  |
| 1～5 | 232 (32.31) | 164 (33.95) | 68 (28.94) |  |  |
| 6～10 | 148 (20.61) | 94 (19.46) | 54 (22.98) |  |  |
| 11～20 | 141 (19.64) | 85 (17.60) | 56 (23.83) |  |  |
| ＞20 | 64 (8.91) | 31 (6.42) | 33 (14.04) |  |  |
| BMI(kg/m^2^),n(%) |  |  |  | χ²=13.40 | **0.009** |
| ＜18.5 | 71 (9.89) | 36 (7.45) | 35 (14.89) |  |  |
| 18.5～24.9 | 453 (63.09) | 304 (62.94) | 149 (63.40) |  |  |
| 25～29.9 | 106 (14.76) | 81 (16.77) | 25 (10.64) |  |  |
| ≥30 | 26 (3.62) | 19 (3.93) | 7 (2.98) |  |  |
| Missed | 62 (8.64) | 43 (8.90) | 19 (8.09) |  |  |
| Smoking, n(%) |  |  |  | χ²=10.28 | **0.001** |
| No | 660 (91.92) | 433 (89.65) | 227 (96.60) |  |  |
| Yes | 58 (8.08) | 50 (10.35) | 8 (3.40) |  |  |
| Drinking, n(%) |  |  |  | χ²=2.16 | 0.141 |
| No | 700 (97.49) | 468 (96.89) | 232 (98.72) |  |  |
| Yes | 18 (2.51) | 15 (3.11) | 3 (1.28) |  |  |
| **Comorbidities** |  |  |  |  |  |
| ILD, n(%) |  |  |  | χ²=4.01 | **0.045** |
| No | 670 (93.31) | 457 (94.62) | 213 (90.64) |  |  |
| Yes | 48 (6.69) | 26 (5.38) | 22 (9.36) |  |  |
| Anemia, n(%) |  |  |  | χ²=3.84 | 0.050 |
| No | 407 (56.69) | 286 (59.21) | 121 (51.49) |  |  |
| Yes | 311 (43.31) | 197 (40.79) | 114 (48.51) |  |  |

**Table S5** Continued

| Variables | Total (n = 718) | RA without osteoporosis (n = 483) | RA with osteoporosis (n = 235) | Statistic | *P* |
| --- | --- | --- | --- | --- | --- |
| Diabetes, n(%) |  |  |  | χ²=5.57 | **0.018** |
| No | 618 (86.07) | 426 (88.20) | 192 (81.70) |  |  |
| Yes | 100 (13.93) | 57 (11.80) | 43 (18.30) |  |  |
| Hypertension, n(%) |  |  |  | χ²=17.95 | **<0.001** |
| No | 538 (74.93) | 385 (79.71) | 153 (65.11) |  |  |
| Yes | 180 (25.07) | 98 (20.29) | 82 (34.89) |  |  |
| Thyroid disease, n(%) |  |  |  | χ²=0.79 | 0.375 |
| No | 640 (89.14) | 434 (89.86) | 206 (87.66) |  |  |
| Yes | 78 (10.86) | 49 (10.14) | 29 (12.34) |  |  |
| Hyperuricemia/gout,n(%) |  |  |  | χ²=0.44 | 0.509 |
| No | 652 (90.81) | 441 (91.30) | 211 (89.79) |  |  |
| Yes | 66 (9.19) | 42 (8.70) | 24 (10.21) |  |  |
| Dyslipidemia,n(%) |  |  |  | χ²=2.01 | 0.156 |
| No | 288 (40.11) | 185 (38.30) | 103 (43.83) |  |  |
| Yes | 430 (59.89) | 298 (61.70) | 132 (56.17) |  |  |
| CVDs,n(%) |  |  |  | χ²=11.73 | **<0.001** |
| No | 591 (82.31) | 414 (85.71) | 177 (75.32) |  |  |
| Yes | 127 (17.69) | 69 (14.29) | 58 (24.68) |  |  |
| Infectious diseases,n(%) |  |  |  | χ²=0.97 | 0.325 |
| No | 586 (81.62) | 399 (82.61) | 187 (79.57) |  |  |
| Yes | 132 (18.38) | 84 (17.39) | 48 (20.43) |  |  |
| **Laboratory examination** |  |  |  |  |  |
| RF(IU/mL),n(%) |  |  |  | - | **0.043** |
| ≤20 | 161 (22.42) | 119 (24.64) | 42 (17.87) |  |  |
| ＞20 | 550 (76.60) | 361 (74.74) | 189 (80.43) |  |  |
| Unrecorded | 7 (0.97) | 3 (0.62) | 4 (1.70) |  |  |
| ACPA(U/mL),n(%) |  |  |  | χ²=0.57 | 0.904 |
| 0～5 | 109 (15.18) | 76 (15.73) | 33 (14.04) |  |  |
| 5.1～200 | 294 (40.95) | 198 (40.99) | 96 (40.85) |  |  |
| ＞200 | 267 (37.19) | 176 (36.44) | 91 (38.72) |  |  |
| Unrecorded | 48 (6.69) | 33 (6.83) | 15 (6.38) |  |  |
| CRP(mg/L),n(%) |  |  |  | - | **0.047** |
| 0～8 | 192 (26.74) | 141 (29.19) | 51 (21.70) |  |  |
| ＞8 | 524 (72.98) | 340 (70.39) | 184 (78.30) |  |  |
| Unrecorded | 2 (0.28) | 2 (0.41) | 0 (0.00) |  |  |
| ESR(mm/h),n(%) |  |  |  | χ²=1.60 | 0.450 |
| Normal | 122 (16.99) | 88 (18.22) | 34 (14.47) |  |  |

**Table S5** Continued

| Variables | | Total (n = 718) | | RA without osteoporosis (n = 483) | | RA with osteoporosis (n = 235) | | Statistic | | *P* |
| --- | --- | --- | --- | --- | --- | --- | --- | --- | --- | --- |
| Higher | 579 (80.64) | | 384 (79.50) | | 195 (82.98) | |  | |  | |
| Unrecorded | 17 (2.37) | | 11 (2.28) | | 6 (2.55) | |  | |  | |
| NPAR, n(%) |  | |  | |  | | χ²=12.81 | | **<0.001** | |
| ＜1.7598 | 359 (50.00) | | 264 (54.66) | | 95 (40.43) | |  | |  | |
| ≥1.7598 | 359 (50.00) | | 219 (45.34) | | 140 (59.57) | |  | |  | |
| WBC(×10^9^/L), M (Q1,Q3) | 6.62 (5.29, 8.29) | | 6.67 (5.21, 8.27) | | 6.57 (5.49, 8.29) | | Z=-0.71 | | 0.480 | |
| RBC(×10^9^/L), M (Q1,Q3) | 4.05 (3.69, 4.41) | | 4.09 (3.74, 4.43) | | 3.94 (3.58, 4.32) | | Z=-3.02 | | **0.003** | |
| HGB(g/L), M (Q1,Q3) | 114.00 (102.00, 124.00) | | 115.00 (104.00, 125.50) | | 111.00 (97.00, 123.00) | | Z=-3.16 | | **0.002** | |
| NEU(×10^9^/L), M (Q1,Q3) | 4.25 (3.08, 5.77) | | 4.17 (3.04, 5.71) | | 4.34 (3.26, 5.82) | | Z=-0.98 | | 0.325 | |
| NEU percentage, Mean(SD) | 64.39 ± 11.54 | | 64.10 ± 11.43 | | 64.98 ± 11.76 | | t=-0.96 | | 0.336 | |
| PLT(×10^9^/L), M (Q1,Q3) | 309.00 (239.25, 385.75) | | 303.00 (240.00, 376.00) | | 313.00 (235.50, 397.00) | | Z=-0.61 | | 0.544 | |
| LYM(×10^9^/L), M (Q1,Q3) | 1.61 (1.21, 2.03) | | 1.61 (1.23, 2.04) | | 1.61 (1.21, 1.96) | | Z=-0.63 | | 0.527 | |
| WBC LYM, M (Q₁, Q₃) | 3.99 (3.12, 5.40) | | 3.94 (3.10, 5.31) | | 4.19 (3.23, 5.60) | | Z=-1.65 | | 0.099 | |
| DDi(mg/L), M (Q1,Q3) | 1.56 (0.67, 3.58) | | 1.36 (0.61, 3.53) | | 1.87 (0.86, 3.85) | | Z=-2.26 | | **0.024** | |
| TC(mmol/L), M (Q1,Q3) | 4.38 (3.74, 5.14) | | 4.37 (3.70, 5.12) | | 4.44 (3.80, 5.21) | | Z=-0.77 | | 0.441 | |
| TG(mmol/L), M (Q1,Q3) | 0.99 (0.74, 1.36) | | 1.00 (0.74, 1.38) | | 0.97 (0.73, 1.28) | | Z=-0.26 | | 0.792 | |
| HDL(mmol/L), M (Q1,Q3) | 1.19 (0.98, 1.46) | | 1.17 (0.97, 1.46) | | 1.26 (1.02, 1.46) | | Z=-1.86 | | 0.062 | |
| LDL(mmol/L), M (Q1,Q3) | 2.85 (2.31, 3.44) | | 2.81 (2.30, 3.44) | | 2.90 (2.34, 3.40) | | Z=-0.38 | | 0.705 | |
| NPAR, M (Q1,Q3) | 1.76 (1.51, 2.03) | | 1.72 (1.49, 1.96) | | 1.88 (1.56, 2.14) | | Z=-3.51 | | **<0.001** | |
| **Medication Use** |  | |  | |  | |  | |  | |
| NSAIDs, n(%) |  | |  | |  | | χ²=1.58 | | 0.209 | |
| No | 240 (33.43) | | 154 (31.88) | | 86 (36.60) | |  | |  | |
| Yes | 478 (66.57) | | 329 (68.12) | | 149 (63.40) | |  | |  | |
| GCs, n(%) |  | |  | |  | | χ²=23.15 | | **<0.001** | |
| No | 467 (65.04) | | 343 (71.01) | | 124 (52.77) | |  | |  | |
| Yes | 251 (34.96) | | 140 (28.99) | | 111 (47.23) | |  | |  | |
| MTX, n(%) |  | |  | |  | | χ²=0.31 | | 0.578 | |
| No | 359 (50.00) | | 238 (49.28) | | 121 (51.49) | |  | |  | |
| Yes | 359 (50.00) | | 245 (50.72) | | 114 (48.51) | |  | |  | |
| LEF, n(%) |  | |  | |  | | χ²=0.52 | | 0.470 | |
| No | 597 (83.15) | | 405 (83.85) | | 192 (81.70) | |  | |  | |
| Yes | 121 (16.85) | | 78 (16.15) | | 43 (18.30) | |  | |  | |
| HCQ, n(%) |  | |  | |  | | χ²=1.48 | | 0.224 | |
| No | 656 (91.36) | | 437 (90.48) | | 219 (93.19) | |  | |  | |
| Yes | 62 (8.64) | | 46 (9.52) | | 16 (6.81) | |  | |  | |

**Table S5** Continued

| Variables | | Total (n = 718) | | RA without osteoporosis (n = 483) | | RA with osteoporosis (n = 235) | | Statistic | | *P* |
| --- | --- | --- | --- | --- | --- | --- | --- | --- | --- | --- |
| SSZ, n(%) |  | |  | |  | | χ²=0.00 | | 1.000 | |
| No | 714 (99.44) | | 480 (99.38) | | 234 (99.57) | |  | |  | |
| Yes | 4 (0.56) | | 3 (0.62) | | 1 (0.43) | |  | |  | |
| IGU, n(%) |  | |  | |  | | χ²=1.13 | | 0.289 | |
| No | 590 (82.17) | | 402 (83.23) | | 188 (80.00) | |  | |  | |
| Yes | 128 (17.83) | | 81 (16.77) | | 47 (20.00) | |  | |  | |
| Tofacitinib, n(%) |  | |  | |  | | χ²=5.14 | | **0.023** | |
| No | 563 (78.41) | | 367 (75.98) | | 196 (83.40) | |  | |  | |
| Yes | 155 (21.59) | | 116 (24.02) | | 39 (16.60) | |  | |  | |
| Baritinib, n(%) |  | |  | |  | | χ²=0.17 | | 0.681 | |
| No | 702 (97.77) | | 473 (97.93) | | 229 (97.45) | |  | |  | |
| Yes | 16 (2.23) | | 10 (2.07) | | 6 (2.55) | |  | |  | |
| TNF-α inhibator, n(%) |  | |  | |  | | χ²=1.48 | | 0.224 | |
| No | 626 (87.19) | | 416 (86.13) | | 210 (89.36) | |  | |  | |
| Yes | 92 (12.81) | | 67 (13.87) | | 25 (10.64) | |  | |  | |
| Z: Mann-Whitney test, χ²: Chi-square test, -: Fisher exact | | | | | | | | | | |
| M: Median, Q₁: 1st Quartile, Q₃: 3st Quartile; SD: standard deviation | | | | | | | | | | |
